# Historical Sketch of the Progress of Medicine for the Year 1810, and from January to June 1811

**Published:** 1811-07

**Authors:** 


Medical and Physical Journal.

VOL. XXVI.] JULY, 1811. [NO. 149.

Printed for R. PHILLIPS, by E. Hemsted, Great New Street, Fetter Lane, London.

### HISTORICAL SKETCH OF THE PROGRESS OF MEDICINE FOR THE YEAR 1810, AND FROM JANUARY TO JUNE 1811.

(No. 5.)

[*To be continued Half-yearly.*]

"A thousand writers, perhaps for a thousand years, have been improving this Art and Profession; and he that industriously studies those authors will, in a short period of time, find out as much as if he had lived a thousand years himself, or employed those thousand years in the study of Physic." FREIND.

"Floriferis ut Apes in saltibus omnia limant." LUCRETIUS.

FOR four years, an historical view has annually been given in this JOURNAL of such circumstances in the science of Medicine, that were of importance sufficient to arrest the attention of the medical Faculty: to flatter into hope, or to depress with apprehension. In the conduct of this detail it was always the object of the writer to give due honour to the science, rather than to encourage the art: to record sound and rational principles, whenever or wherever they were found: to select and arrange interesting facts and ingenious observations; to excite a steady attention to the laws and operations of Nature, and to advise, the student at least, neither to disdain nor to neglect the collateral and auxiliary sciences.

The change which has taken place in the management and œconomy of the *Medical and Physical Journal*, will in no wise alter the principles or objects of this Report, or affect its usual detail, except, that instead of being published annually, it will, in future, be given every six months, and necessarily become a species of *prolegommena* to each volume.

IT has been thought a propriety to begin a view of the Progress of Medical Science, with an account of the efforts which occasionally are made for improving the respectability of that science, and the consequent good of mankind: to shew the progress of these efforts; to point out the obstacles they they have met with, and the objections to their principles, or their operative detail.

During the period under consideration, the persevering spirit of Dr. Harrison, still pursuing its object against the coldness of neglect, the hostility of interest, the argument of knowledge, and the jest of wit, contends for a specific mode of amending the condition of the medical profession, and for giving to it a CODE, to be rendered permanent by an act of the legislature; which shall, in future, determine the essential qualifications required in those who enter on the exercise of the medical art; which shall distinctly point out certain regulations applying to those who may be deemed qualified for this important office, and detailing other circumstances calculated, more or less, to alter the condition, legal or social, of persons employed in every branch of the science of healing. The advance so far, of Dr. Harrison's project, as to indicate an immediate application to the Commons House of Parliament, was not beheld by the Faculty without sensations of a very opposite and contradictory nature. These sensations drew their character from circumstances in the classes of medical persons among whom they arose. When Dr. Harrison published the outlines of his intended Bill, much was said, and it was proper that much should be said upon it; and that the operation of its principles, and their final effects, should be deeply investigated, and fully ascertained. Of the mass of heterogeneous opinions, which were either whispered in the seclusion of private society, or boldly claimed the public ear, a solution might be offered. It was obvious to remark, that those who were in possession of exclusive privileges thought this Bill was not called for, or that its clauses went too far. Those who actually were, or thought themselves inequitably kept out of certain rights, held, or as they said, usurped by some privileged orders, though they approved of a change, pronounced Dr. Harrison's Bill to be imbecile and inefficient: in short, for them, it did not go far enough. Another class, enjoying no corporate privileges, and expecting none, may be considered as neutrals. If this Bill gave them nothing, it took nothing from them, for its principle was to leave the present race of medical men undisturbed: and they were content, whether the project was fated to success or to defeat. It would argue a want of candour to deny, or not to state, that there were to be found among the Faculty many individuals who were warmly solicitous to meliorate the science of medicine, to improve the condition of those properly employed in it, to suppress cheats and impostors, to protect honest merit, and to reward talent. To see the healing art rescued from the mean and degrading circumstances cumstances in which it was often found, was the laudable object of these individuals; to place it in a station which its importance demanded, and to protect it by wholesome and rational laws which its usefulness deserved. To them it was not important from whence the desired reform came; and though they might not assent to every iota in Dr. Harrison's Bill, they could not withhold their approbation of his public spirited perseverance, and did not object to him because he resided in the fens of Lincolnshire, or was not a fellow of the Royal College. Whatever may have been said about the inherent desire for change in the human mind, it seems a proposition equally defendable, that an attachment to old customs and long established habits affords more than a counterpoize to the principle of mutability. It is not certain that this attachment to customs consecrated by ancient usage, did not influence the opinions and feelings of many well-meaning members of the medical faculty, and lead them to resist the purposed improvment, upon the principle that the change of established forms is always dangerous.

> "Omnia Fata laborant

> Si quidquam mutare velis; unoque sub ictu

> Stat Genus Medicum."

Those who held privileges, and those who held none; those who believed themselves deprived of their rights, and those who feared a change upon the ground that all change was dangerous, united into a phalanx compact, formidable, and impenetrable. Against this host, drawn together by various motives and feelings, but all looking to particular interests, fearing the loss of some good already possessed, or apprehending the demolition of some expectancy, it would have been unwise to contend; and Dr. Harrison, judiciously perhaps, suspended his projected plan of reform, to resume it, however, if the past is to predict the future, at some more auspicious period.

It will be right to state the principle, and the prominent features of this Bill, which had penetrated into every rank and degree of the children of Esculapius; and spread alarm from the college chief to the lowest medicaster, the pro bono distributer of salutiferous pills and antibilious bolusses.

Under the operation of various irritations, it is creditable to the understanding and to the temper of Dr. Harrison, that he has proceeded with coolness and moderation; that he has replied to suspicion, to wit, and to argument, with temperate explanation, and a professed desire to profit by observations of every quality and tendency. Under these dispositions, and assisted by persons learned in the law, it appears that the principle of the projected Bill was devised, and its

 clauses clauses manufactured. In the principle itself may be observed a willingness to accommodate and to temporize, an anxiety to lead the Faculty on into a sufferance of laying the foundation of material changes, to allay the fears of government as to productive taxation, and to cajole into security the hydra-headed monster quackery. In its object, however, it has entirely failed. What was intended to accommodate all, has pleased none. Its having no retrospective operation was a marked trait in its character. All those in actual practice at the time of passing the Bill, were to remain in undisturbed possession, whether their emoluments arose from donations in the form of fees, or were exacted from the public under the authority of a patent; whether they came under the denomination of apothecaries, a useful and frequently intelligent body of practitioners, or caught the unwary in the cunning evolutions of diurnal paragraphs. Thus it seemed to establish the impudent and unlettered empiric on as firm a basis, with a right to practice as efficient, as that which it gave to the learned and scientific graduates of our universities. It specifically determined also to leave untouched the great evil, advertised nostrums; and it allowed the lowest charlatan to practise under the licence of men as ignorant, on the subject of medicine, as themselves*. Should Dr. Harrison no more revive his project, though from internal evidence, the contrary conclusion has been made, he may claim the merit of having awakened attention to a subject of mighty importance to society: and if he has had the misfortune to have called forth untimely the crude conceptions of a rabble of empirical adventurers; he has also had the happiness to elicit the observations of men of serious investigation, cautious research, and of eminent talents. Throughout a trying period he has not departed from the urbanity of a gentleman; but aware of the difficulties which lay before him, he has endeavoured to remove them by making his views palatable; and thus by conceding to the prejudices of some, and admitting the interests of others, he has rendered his project, perhaps, emasculate and inefficient. He has not lost sight of his principle, but he has erred in the selection of his means. More than a century ago the ingenious physician and deep calculator, Sir William Petty, remarked, "as for Physicians, it is not hard, by the help of observations which have been lately made upon the Bills of Mortality, to know how many are sick in London by the number of them that die, and by the

* For a statement of the clauses of this Bill, see Med. and Phys. Jour. vol. 24, 495—and for observations in opposition to, and in support of its principles, vol. 25. 109, 216, 402, and 492.

proportion proportions of the city to find out the same of the country; and both by the advice of the learned college of that faculty to calculate how many physicians were requisite in the whole nation; and consequently how many students in that art to permit and encourage: and lastly, having calculated the numbers, to adaptate a proportion of chirurgeons, apothecaries, and nurses to them, and so by the whole to cut off and extinguish that infinite swarm of vain pretenders unto, and abusers of that godlike faculty, which of all secular employments our Saviour himself, after he began to preach, engaged himself upon."

To understand fully how requisite reformation in the privileges and claims of medical practitioners may be, it will be necessary to go into the history of the progress of the art: to ascertain how, under certain circumstances it has flourished, and how, under others, it has been depressed: to mark what has been favourable to its melioration, and what has retarded its improvement: to determine whether acts of legislatures have ever assisted the progress of science, or whether the investigations of philosophy and the study of nature have not been most successful when left unshackled and free from the restraint of laws. The reformer should be able to appreciate the wisdom of ages, and to apply the acts of distant periods to circumstances of the passing moment: from the multitudinous instances of wisdom and of error to select, analyze, and condense. Thus prepared, he has data wherewith to lay the foundation of reform. Nor are there wanting histories and documents to aid this species of research. From Le Clerc, Freind, Sprengell, and Cabanis, the opinions and practice of remote and present times may be collected. And to these may now be added the labours of Dr. Millar of Glasgow. A series, or part of a series of "Disquisitions" on the history of medicine by this gentleman has been recently added to medical literature*. They trace the history and

* These disquisitions, the author says, owe their existence partly to some singular traits which he fancied he had discovered in the medicine of the early Greeks, and which he found attached no less strongly to the art, as practiced among other rude and semi barbarous tribes; and partly to the new light thrown on the primitive physic of mankind by the extraordinary advances, attained to of late years, in Sanscrit Literature. From this last source it is learned beyond the possibility of doubt, that long previous to its cultivation in Europe, very considerable progress had been reached by the science of healing, in Hindustan, and that a remarkable coincidence is to be discerned between its precepts, as inculcated by the Brahmans, and as promulgated of old by the respective hierarchies of Iran and Egypt, more especially the latter: but though from this and other evidence, it is equally certain, that by the efforts of these progress of this branch of science from the most distant periods of time, before the rise of letters and philosophy in Greece, from the primeval Chersonese through the traditionary and heroic ages of the Grecian history, to Egypt and India. In this ingenious work, a part of which seems now only to appear, in addition to the curious research of the text, is found a body of annotation, comprehending an extensive mass of materials, illustrative, not only of the history of medicine, but of man.

In Anatomy, though the foundation of all medical and chirurgical knowledge, no novelty has occurred within the last eighteen months. Perhaps the minutiæ of this art being pushed near to its acmé, has left little to be discovered. Consisting altogether of facts, it has its limits. When every fibre has been separated, examined, and explained, the Anatomist, like Alexander, may weep for new worlds of animal existence. To the Physiologist this state of exhaustion will never happen. The stores of the imagination are perennial: if one series of opinions or suppositions are overthrown or exhausted, others arise. The Physiologist resembles that great poet, who, "exhausted worlds and then imagined new." And his science, on many occasions so nearly allied to invention, will seldom permit any period to pass totally barren of incident, fact, or hypothesis, intended to explain some action or train of actions belonging to animal or vegetable life; forms of being which mutually illustrate each other.

For many ages, the doctrine of generation, still perhaps the most abstruse of physiological speculations, turned on a circumstance so contrary to all truth, and so opposed to all analogy, though with the appearance of being founded upon observation, that in this age of philosophical illumi-

---

these learned bodies, a nearly regular system of physic had been erected, at a very remote era in the East, yet to commemorate its details, or appreciate its merits, has never become the task of any historian of medicine. In this new field of medical archæology Dr. Millar has ventured to labour. By what is now published, an attempt is made to supply the chasm, as far as Egypt is concerned: in a subsequent volume it is intended to investigate the condition of the healing art, such as it may be found to have flourished in the other two oriental Monarchies, no less distinguished for their early proficiency in arts and science, namely, Hindustan and Iran. To this it is intended to subjoin in an Appendix, a Treatise on the Physic of the antient Jews. We congratulate the medical profession, on the prospect of an interesting portion of the history of the art being supplied by a person apparently so well qualified for the task as Dr. Millar: and we trust no appearance of neglect will occur to prevent the completion of what we consider to be an historical desideratum.

nation, nation, the only feeling that remains respecting it, is asto-nishment. The hypothesis of equivocal generation, which existed so long and was to pertinaciously adhered to, could not be supported against even the dawn of experimental philosophy. The investigations of Harvey, Malpighi, Swammerdam, Valisneiri, Ray, &c. extinguished this absurdity; and the aphorism *omnia ex ovo* is as fully established as any precept in Physiology. To support this proposition, founded as it is on sound reason and unwearied inquiry, an infinite multitude of experiments have been made and numerous observations recorded, which lie dispersed in a vast number of volumes. It chances to be the fortunate lot of but few to explore the source, or follow the current of this flood of laborious and learned investigation: the naturalist and the physician are therefore indebted to Dr. Paris for a "Memoir on the Physiology of the Egg," which has been read before the Linnean Society of London; and which seems to have condensed into an historical and philosophical essay, the whole of the facts and deductions of the ovarian Physiology. To this detail Dr. Paris has added, it is understood, several new facts, which not yet being fully before the public, cannot, without improper anticipation of their author, be particularly stated.

The *prima stamina* of many ingenious works, which have subsequently been matured into standard productions, first appeared in this Journal. It cannot be necessary to enumerate these, but it is proper to notice a most ingenious physiological inquiry by Mr. Smith of Bristol, under the denomination of a "Theory of Sensation." (Jour. 25. 467.) This speculation, when it has received the finishing touches of its author, promises, from the ability with which it commences, to rival in precision and practical facility the theory of Darwin. It affords a peculiar gratification to the Editors of the Medical and Physical Journal, to observe how frequently it has been the arena where native talent has first manifested itself, and where genius has ventured its earliest flights.

The illustration that general nature receives from analogies that the study of parts develope, has never been more fully admitted than in the present day. The similarities and coincidences observed to take place among forms of being, apparently, far removed from each other, explain, very fully, that the whole of animated nature is governed by a system of laws as uniform in their operation as their influence is extensive. This fact has received a striking exemplification by Mr. Knight, in observations on the progress of life and the approach of old age and final decay in trees. "Whatever difference difference exists between the functions of animal and vegetable life, there is," says Mr. Knight, "an obvious analogy between some of the organs of plants, and those of animals; and it does not appear very improbable that the correspondent organ, in each, may first fail to execute its office." The analogy between the leaves of plants and the lungs of animals is considered to be very close, and that in both, the inability in this organ, whether leaves or lungs, to make that change in the circulating fluid which is essential to health, occasions decay and final destruction to the individual in which the respiring organ fails to perform its office. Experiments satisfied Mr. Knight that the debility and diseases of some old varieties of fruit trees, did not originate in any defective action of the bark or alburnum, either of the root or of the stem and branches, but in the leaves and succulent annual shoot. He observed also, that grafted trees, of old and debilitated varieties of fruit, became most diseased in rich soils, and when grafted on stocks of the most vigorous growth; and he was induced to conclude, that in such cases more food is collected, and carried up into the plant, than its leaves can prepare and assimilate, and that the matter thus collected, which would have promoted the health and growth in a vigorous variety, accumulates, and generates disease in the extremities of the branches and annual shoots of the old and debilitated; and that the diseases and weakness of old age in trees, arises from the want of power to produce leaves which can efficiently execute their natural office; and to some consequsnt imperfection in their circulating fluid. If these opinions be well founded, and the leaves of trees be analogous to the lungs of animals, is it not probable that the natural debility of old age in trees and animals may originate from a similar source? If it is a fact, that not only man, but those domestic animals, as well as others, longest retain their health and strength, and best bear excessive labour and insufficient food, in which the chest is most deep and capacious, in proportion to the length of current the circulating fluid has to run; and that those trees longest retain health and vigour whose foliage longest continues in the perfect exercise of this pulmonic function, an additional analogy is established between animal and vegetable life.

If the medical student desires to possess the enlarged views that constitute the accomplished and philosophic physician, he must study nature, in all her departments, with unceasing diligence. There is not a fact in the whole circle of her history, which may not be found valuable, on some occasion, in the practice of his art. The analogies that exist among all beings, the influences that reciprocally operate upon each, the the explanation that is given to some by the peculiarities of others, the resemblance and the diversity of their diseases, the remedies they afford or the poisons they inflict, all are objects of interest and importance. Not a leaf nor a blade of grass, a stone or a metal; not an animated being from the acarus to the elephant, but may afford instruction. Not an individual among all these but tends in some degree or mode to illustrate some other creature, or to explain some law in nature; but presents a study for man, and gives the means of enlarging his views, and refining his understanding. Yet where is the mind of capacity to comprehend the laws that govern, or the peculiarities that distinguish these miriads? If those who pursue the study of nature despair, let them remember the toils as well as the talents of those who have gone before to direct them. The comprehensive mind of Bacon, the minute exactness of Spallanzani, and the systematic genius of Linneus, have so far unfolded these intricacies, and pointed out the road to natural truths so clearly, as to leave, comparatively, but little of mystery. And since the time of Linneus, in the particular department to which he almost gave birth, certainly direction, much, by a thousand others, has been discovered, explained, and accomplished. The daring enterprize, the correct observation, and the skill which was seen in the pupils of the Swedish naturalist, in Kalm, Osbeck, Hasselquist, Sparmann, Solander, and many others*, who were employed to visit remote countries, have been revived in Humboldt, whose travels in some parts of South America rival, if they do not surpass, the most illustrious of the Linnean school. This person, a Prussian gentleman of fortune, under every favourable circumstance resulting from his own acquirements in all the branches of knowledge which could be useful in such an undertaking, having for his companion M. Bonpland, and countenanced, or even authorized by the Spanish government, began in 1799 an expedition to Spanish America, in which he was engaged

* It must not be forgotten that Peter Löfling, the favourite pupil of Linné, was selected to travel through the different provinces and settlements of the Spanish government in South America. But scarcely twelve months had he been in America before he was seized with a tertian intermittent, from the consequences of which he died in 1756. The substance of his travels was published by Linneus, at Stockholm, in 1758, with title of Iter Hispanicum, eller Resa til Spanska Länderna uti Europa och America, &c. &c. But this did not, in any degree, anticipate the exploration of Humboldt. Among them who have encouraged by their example, and patronised by their liberality, those who have sought to explore nature in savage lands and remote regions, the President of the Royal Society is pre-eminent.

till 1804. The facts and the final result of this interesting expedition are intended to be given to the public in a work of great scientific splendor. The part of this which is already published presents many new and curious facts in the history of man, in the lower orders of the animal scale, and of vegetables and minerals, as well as in geology and philosophical geography.

The effect of climate on the human frame has always been an object of solicitude. The details of Humboldt furnish some interesting facts on this subject. It appears from authentic documents, that there is a rapid increase of population in New Spain. The proportion of births to deaths throughout the kingdom of Mexico is as 170 to 100: and in some parts of the table land (a district of land among the mountains of Mexico elevated from 6000 to 8000 feet above the sea) the proportion was as high as 250 to 100: but at Panuco, on the coast of the North Sea, it was as low as 123 to 100. This difference arises from the greater salubrity of the table land in the centre of New Spain, compared with the low marshy lands on the coast. The salubrity of the tropical climates depends more on the dryness of the air, than on any of its other sensible qualities. The burning province of Cumana, the coast of Coro, and the plains of the Caraccas prove, Mr. Humboldt observes, that excessive heat alone is not unfavourable to human life, and that in very hot and dry countries mankind attain to a greater age than in the temperate zones. At Lima a Peruvian Indian died at the age of 147, having been married for 90 years to the same woman, who had lived to the age of 117. Wherever the air is moist as well as hot, the climate is exceedingly unwholesome. It is so upon the north coast of Mexico, from the mouth of the river Alvarado to the river Tampico, and the plains of New Santandon. The south coast is equally unhealthy, from San Blas to Acapulco. The combination of heat and moisture in the atmosphere, in like manner, renders the coast of the Caraccas unwholesome, from Barcelona to Puerto Caballo. This is in direct opposition to the influence of the atmosphere in the temperate and cold countries; in them a cold and dry atmosphere is most destructive to life. The table land, the salubrity of which Mr. Humboldt states to be so remarkable, rising, it was before observed, from 6000 to 8000 English feet above the sea, forms a continuous plain, comprised between 18° and 40° of latitude, and extending in a straight line from Mexico to Santa Fé, a distance of about 500 leagues. The slight ridges which interrupt the absolute uniformity of this plain are seldom more than from 600 to 800 feet above the valleys which they separate. Some of the mountains, indeed, indeed, which rise from its surface, are of colossal magnitude; but the tops of four only are covered with perpetual snow. The highest, called *Popocatepetl*, is 17,000 feet above the sea. The table land of Mexico gradually declines in elevation toward the north, but with so gentle a descent that a carriage may pass from Mexico to Santa Fé in New Mexico. At Santa Fé de Bogota, Quito, Caxamoria, and various other parts of South America, the table land is of the same elevation as in Mexico; but it is no where of the same extent. Forty square leagues form the longest surface that any where presents itself united. These portions of elevated land are insulated and divided from each other by transverse valleys, some of which are 4600 feet in depth; and they have, consequently, the appearance of islands, surrounded and separated by a sea of air. These insulated spots enjoy a salubrious climate and fertile soil; but they are cut off, in a great measure, from all intercourse with one another, or with the rest of the world. The descent from them is painful and fatiguing; and their inhabitants, accustomed to the pure and cool air of the mountains, become sickly and faint, when exposed to the suffocating heat of the valleys. The more elevated plains of the table land are arid, barren, destitute of trees, and covered with a saline efflorescence: but a great part of it is extremely fertile. The nature of its productions varies with its elevation above the sea. Sugar, cotton, cocoa and indigo, do not succeed at a greater height than 2000 or 2800 feet above the sea. European wheat begins to be raised at 4800 feet, and ceases at about 9000. The banana tree gives hardly any fruit at a greater height than 5000 feet. The Mexican oak grows from 2400 to 9500, and pines from 5500 to 15,000 feet above the sea.

This general and enormous elevation of the soil, is one of the most important features of the American continent. In Europe the highest tracts of cultivated land seldom rise more than 2000 feet above the sea. But in the Peruvian territory extensive plains occur at an altitude of 9000 feet; and three fifths of the viceroyalty of Mexico presents a surface of half a million of square miles, at an elevation from 6000 to 8000 feet, equal to that of the celebrated passages of Mount Cenis, of St. Gothard, or of the Great St. Bernard. The mountains themselves tower to most enormous and sublime heights. The extreme top of Chimborazo was ascertained to be 21,340 feet above the sea. Mr. Humboldt and his adventurous companions reached the height of 19,300 feet, the highest spot ever trod by man. At this elevation the air was reduced to half its usual density; blood oozed from their eyes, their lips and their gums.

The equatorial regions of America, possessing, in consequence of their vast range of elevation, every possible degree of temperature, concentrate all the diversity of the vegetable tribes. From the shores of the Atlantic to the heights of the Andes, the different kinds of plants follow each other in almost regular succession. Ascending these mountains from the lower valleys, chesnuts, beeches, oaks, and pines successively appear; and the last advance, till they become stunted, and gradually disappear, not far from the verge of perpetual snow. To trace the geography of plants in the low grounds of Europe, is rendered impossible by the activity of cultivation; but in these boundless deserts, each species still occupies its own territory.

To follow M. Humboldt throughout his interesting details, and it is difficult to forbear, would require a volume. The observations on the genus Cinchona must not, however, be omitted. The trees which furnish the Peruvian Bark are scattered along the chain of the Andes, over an extent of two thousand miles, at an elevation from 2,300 to 9,500 feet. The *lancifolia* and *cordifolia* prefer the plains; the *oblongifolia* and *longiflora* occur somewhat higher; but the noted *quinquina* of Loxa, and which Mr. H. proposes to call *Cinchona condaminea*, grows at a height of from 6,250 to 8,300 feet, where the mean temperature varies between 59 and 62, on a bottom of micaceous schist in the woods of Caxanuma and Uritucinga. This shrub forms one continued forest on the eastern declivity of the Andes, as far as the province of Jaen, and the hills above the river Amazons. Bark of a similar quality is obtained from very distinct kinds of the cinchona, in the same manner as the *caoutchouc* is procured from the inspissated juice of a variety of vegetables, from the *ficus*, the *hevea*, the *lobelia*, the *castilloa*, and several species of the *euphorbium*. The station and climate of many other plants which exist on the vast sides of the Andes, are marked with scientific precision. Some occur, as the *wintera* and the *escallonia*, at the altitude of 9,200, to 10,800 feet, and form scrubby bushes in the cold and moist climate of the *Panamas*. Above the height of 10,500 feet, the arborescent vegetables disappear. The alpine plants occupy an elevation from 6,500 to 13,000 feet: there grow the *gentians*, the *stœlina*, &c. The grasses appear at a height from 13,500 to 15,100 feet. In this zone, where snow falls at times, the *jarava*, and a multitude of new species of *panicum*, *agrostos*, *avena*, and *dactylis*, cover the soil with a yellow carpet, which the inhabitants call pajonal. From about 15,000 feet, to the boundary of perpetual congelation, the only plants visible are are the lichens, which cover the face of the rocks, and seem to penetrate under the snow.

Of the animal kingdom many circumstances are related of importance and interest. Man, here, as in every other region, presents the primary object of attention and regard. His form, his colour, his moral and social character as appears in these surprizing regions, are delineated with spirited exactness. The Mexican Indian is grave, melancholy, and silent, unless when under the influence of intoxicating liquors. He affects an air of mystery in the most unimportant transactions; and no expression is to be seen in his countenance, of the most violent passions that agitate him. Like all enslaved nations, he is obstinately attached to his antient customs, manners, and opinions; and, though converted to christianity, his change of religion is more apparent than real. He seems to be destitute of imagination, and to have little feeling; but, when properly educated, he shews an aptitude for learning, a clear head, a logical and acute understanding. He has a particular turn for painting and carving in wood and stone; but even in these arts, he displays rather a talent for imitation, than genius for invention. His national music is mournful and melancholy; and in his national dances the men only are performers. Among the various nations, tribes, and names, that range over this vast continent, the *Guaranis* presents a new picture in the history of man. It would be a want of taste and feeling not to admit, into the pages of this Report, the exquisite picture of these people and the accompanying scenery, as drawn by an admirable journalist. "As the summer advances, the low plains on the coast of America become parched with excessive heat. The grass withers to the roots, and the soil turns hard and baked. The cattle, enveloped during the day in dust, run panting with oppressive thirst. The more sagacious mule, with his hoof cautiously thrusting aside the prickles of the water-melon, sucks a refreshing beverage. But the cries and frightful shrieks of the larger apes at last announce the approaching rains. Incessant torrents descend. The crocodile and the boa, long concealed in a torpid state under the hardened mud, now raising their horrid fronts, burst, with sudden and tremendous noise, from their tombs. The rivers soon overflow their banks, and sweep the surface with wide inundation. One sheet of water covers the whole delta of the Orinocco. In the midst of this aquatic scene, lives in peace the unconquered nation of the *Guaranis*, who nestle among the tops of the *mauritias*, or fan-leaved palm, in extended hammocks, which they construct with netting made from the fibres of the leaves, and line partly with with mud. On such humid and pencile floors, the women light their fires and cook their vegetable diet. The tree to which each family is attached furnishes its whole subsistence. The pith of the *mauritia*, resembling sago, is formed into thin cakes; and its scaly fruits, in the different stages of their progress afford some variety of wholesome food. Palm-wine supplies an agreeable and refreshing drink, and may even procure that state of intoxication which is the elysium of the savage."

The influence of climate on the colour of the human race has been the subject of great discordance of opinion, and of both religious and philosophical controversy. The permanent or the accidental source of this quality, was a point on which it had been attempted to decide whether the whole of the human race came from one stock, or had arisen from a diversity of parents. The facts stated by Humboldt are of a nature, perhaps, to raise doubts on very serious questions. The facts themselves, however, are all that can be touched here. Climate, this traveller observes, which has such influence on the European race, has little or no effect on the complexion of the Indians. Some tribes are darker than others; but the difference is quite independent of climate. Those who live on the Rio Negro are darker than those who inhabit the banks of the lower Orinocco, though they live in a much cooler temperature. Near the sources of the Orinocco, there are tribes of a light complexion, surrounded by others of a much more swarthy colour. The Indians who live in Chili and on the tops of the Andes, are as dark as the inhabitants of the plains, though the former are cloathed and the latter go almost naked; and those parts of their body which are constantly covered, are not lighter than those which are continually exposed to the sun and air. The Mexican Indians, though they inhabit the same climate with the natives of Quito, are of a darker colour; and those who live as far north as the river Gila, are swarthier than the inhabitants of Goatemala. Contrary to the information obtained by M. Volney concerning the North American Indians, Mr. Humboldt has observed, that in Mexico, Peru, Quito, Caraccas, and other provinces of Spanish America, the children of the Indians are copper-coloured from the moment of their birth; and the Caciques, who are commonly clothed, have all parts of their body of the same copper-colour, except the palms of their hands, and the soles of their feet. It appears, therefore, that the copper-colour of the Aborigines of America is independent of climate; and the same is probably true with respect to the darker complexion of the Negroes and Hindoos.

Of

Of the inferior orders of the animal creation, this ingenious traveller relates numerous facts as curious as they are important. But while he brings us acquainted with many new and surprizing circumstances, he corrects the fabulous tales related by the credulous and ill-informed. It has generally been believed that there existed among the dreary wastes and wide solitudes of the Andes, a fierce and powerful bird of prey, of a size never seen in the old continent, and which with singular incongruity, had been confounded with the Gryphon of the antients.* The touchstone of truth has, however, reduced the *Condor* to the size of the Alpine vulture of Europe: its extreme length being only three feet and a half, and its breadth across the wings nine feet. If truth has diminished its size, it has also determined some of its properties, certainly not so distinctly known before Humboldt's expedition. Its fierceness, cruelty, and voracity, are established, and its wonderful power of wing ascertained. Estimating from very probable data, this bird skims whole hours at the height of four miles; and in an instant it can dart from the chill region of mid-air to the sultry shores of the ocean. Among the extraordinary forms and qualities of animal life, nothing in the discoveries of Mr. Humboldt strikes with more astonishment than the *Gymnotus electricus*. But for a detail of this, though with reluctance, the *Tableau Physique* must be referred to.†

In addition to the preceding curious facts, M. Humboldt has given a view of the diseases of Mexico, (Med. and Phys. Jour. vol. 25. 239.) and a statement of the peculiarities of its atmosphere, as developed by the thermometer and barometer, with general meteorological details.

The vast unexplored continent of Africa probably contains, within its extensive wilds and forests, facts in the history of nature no less surprizing than those which have been related, by Humboldt, of the new world. During the last year a peculiarity in the human form, connected with this continent, and which was thought to have been an invention of Vaillant's, has been verified. That lively traveller described a race of people under the name of Houzoanas, who lived remote from the Cape near to Caffraria, the females of

* It is remarkable that the Gryphon of the antient world should have been made the guardian of gold mines, and that the Condor of the new, its conjectured prototype, should exist where this precious metal is most abundant. The Spaniard has been as eager as the Arimaspian to "purloin the guarded gold," and has more successfully eluded the winged centinel.

† This report is much indebted to a masterly sketch of Humboldt's travels inserted in the Edinburgh Review.

which which had the extraordinary and uncouth peculiarity of *nates* extended to a most enormous and unnatural size. That M. Vaillant was correct in his relation has been proved by one of these females being brought, in 1810, to London. (Med. and Phys. Jour. 24, 385.) To the account of this woman in the place referred to, the following peculiarities are added from the information of a gentleman well known to science and literature. The bony structure of the pelvis has no part in producing the protuberance of the *nates*, for the sacrum has no greater degree of projection than in the European female: the protuberance itself is not muscular fibre, but adipose substance. The arch of the pubis is singularly flattened or depressed; there is little or no mons veneris, but that part which answers to this elevation in the females of other countries, has scattered over it small tufts of woolly hair. The external labia are very thin, and entirely without adipose substance. The nymphæ are much elongated, extending more than four inches, and seem to be a continuation of the external labia. The præputium clitoridis is much longer and thinner than in the European female, and from the defective state of the labia pudendi, appeared to project like a large mass of fleshy substance. The vagina is remarkably short; and the breasts are large and pendulous. A very dark areola covers nearly half the mamma, giving it a most disgusting appearance. The profile of the abdomen resembles, very nearly, that of the ourang outang: it is compressed at the scrobiculus cordis, but from the umbilicus downward, forms an ugly pendant projection. From the absence of mons veneris, and from the projection of the abdomen, the pudendum is perfectly concealed from view. The face of this female has a specific character. Though it has the flattened nose and projecting maxilla of the negro, it is not the face of the negro. A part of its character is derived from the uncommon distance at which the eyes are placed from each other.

The taste and enterprize of an individual has added considerably, from the beginning of 1810, to the stock of Natural History in this country. The museum of Mr. Bullock is a valuable depository of the rarest specimens in this science; preserving with characteristic truth, quadrupeds, birds, fishes, reptiles, and insects. The *Camelopardus giraffa*, within the period above mentioned, has been added to this collection: it is in a fine state of preservation, and measures seventeen feet nine inches in height. So little is now known in Europe of this extraordinary animal, that some persons of education and high station in life have doubted if this specimen be not factitious. The animal has, however, been often seen alive, stalking across the forest glades of the

African

African wilderness; and a living one was lately shewn at the Cape.* The Giraffe was not such a stranger to the Romans. In the collection of animals prepared by the younger Gordian for his triumph there were ten Camelopards. The sacred Ibis, the *Buceros Africanus*, almost an hundred species of *Trochilus*, (Humming bird), the whole of the known individuals of the genus *Paridisea*, (bird of Paradise) adorn this museum by the singular elegance of their plumage, or illustrate by their rareness the page of natural history. This collection is not confined to the beings which now inhabit this earth: there are in it many specimens of the remains of creatures, which at some unknown period had existence here. Among these there is part of the foot and a claw of an animal of the order *Feræ*, of tremendous size. Imagination recoils from a tiger whose paw, when expanded on its prey, covered a superficies of twelve feet. To correspond with the parts here deposited, such indeed must have been the size of the foot of this animal. This extraordinary fossil bone was discovered near the banks of one of the American rivers, where such remains are not uncommon. But a few years since there was discovered near the Missouri, a space of a quarter of a mile in extent, wholly covered with these enormous bones, to the depth of six feet. A person employed there in searching for mines, engaged to complete a skeleton of the mammoth, fifty-four feet in length, and twenty-two feet high. The pages of this Journal (vol. 25. p. 97.) have recorded an instance, illustrated by the scientific observations of Mr. Brooks, the Anatomist, of the fossil remains of a large animal of the Lacerta genus; so perfect as to prove that it could not belong to any individual of that genus now known to exist. Of all the objects which nature presents to the observation of man, the fossil remains of animals now extinct are the most extraordinary and incomprehensible. The existence of races of beings of enormous size, and which are now no where to be found alive, rests on evidence so demonstrable and positive, that M. Cuvier, from the facts which prove that they once lived on this earth, has been able to distinguish twenty-three species of these incognitæ.†

---

* The Girafle in Mr. Bullock's Museum, was shot in the interior of the territory of the Cape, by the Rev. Mr. Evans, an African Missionary.

† The species enumerated by M. Cuvier, are, 1. The Siberian animal which affords fossil ivory. 2. The mammoth, differing from the former chiefly in the size and points of its grinders. 3. The long-headed rhinoceros. 4. The megathereum. 5. An extinct species of large bear. 6. Another species of bear. 7. A carnivorous animal be-

 tween

The practice of philosophical chemistry makes a conspicuous part of modern science. The comprehensive views, and the ardent research of the chemist; his discoveries and his explanations of the mysterious operations of nature, place him very high among those who are devoted to intellectual pursuits. It is to be lamented, however, that still his art is involved and uncertain; and, notwithstanding the advances of the preceding twenty years, data are yet wanting wherewith to form a permanent system. But the labour of experimental investigation, and the fortune of discovery, goes on, to terminate, perhaps, in that developement of principles, which may furnish a sure basis for a perfect theory. Mr. Davy is pursuing his interesting inquiries on the application of electricity to chemistry. The principal part of his late Bakerian Lecture consists of new experiments on the metals of the fixed Alkalies. When Mr. Davy published his brilliant discovery of *Potassium* and *Sodium*, an epitome of the facts and results was inserted in a former number of this Report. These discoveries were of a nature to impress the philosophical chemist; and it could not be doubted but various opinions would arise upon them. "The generality of enlightened chemists," says Mr. Davy, "have expressed themselves satisfied with the experiments, and the conclusions drawn from them: but as usually happens in a state of activity in Science, and when the objects of enquiry are new, and removed from the common order of facts, hypothetical explanations of the phenomena have been given, different from those I adopted." M. M. Gay-Lussac and Thenard suppose potassium and sodium to be compounds of potass and soda with hydrogen; a similar

---

tween the wolf and hyæna. 8. A creature akin to the mouse, whose horns measure 14 feet from tip to tip. 9. The great fossil tortoise. 10. The Maestricht crocodile. 11. A sort of dragon. 12. An unknown kind of reptile, or cetaceous animal. 13. That animal, the teeth of which when impregnated with copper, form the occidental turquoise. 14. A tapir. 15. Another tapir of gigantic size. 16. A species of hippopotamus, about the size of a hog. 17 to 22. Fossil skeletons of unknown species, between the rhinoceros and tapir, from the plaister quarries in the neighbourhood of Paris. 23. A species of crocodile, resembling that of the Ganges. Beside these the earth contains many more parts of skeletons of which M. Cuvier speaks with less certainty. Some of these resemble the bones of the tyger, the hyæna, and the fallow deer. Others of more uncertain character have been found near Verona, in the rock of Gibraltar, in the vicinity of Dax, near Orleans, near Aix and Cette, in the islands of Dalmatia, and in the peat mosses of all Europe and Asia. The third volume of Mr. Parkinson's "Organic Remains" about to be published, may be expected further to illustrate this interesting, but obscure subject.

opinion seems to be entertained by M. Ritter. M. Curadau affects to consider them as combinations of charcoal and hydrogen with the alkalies; and an enquirer in Nicholson's Journal regards them as composed of oxygen and hydrogen. In substantiating his claim to opinions on the nature of potassium and sodium, which had been formed on the basis of experiment, Mr. Davy examines the preceding conclusions as far only as they are connected with and arise out of experiments, and leaves untouched those which are merely hypothetical. After an elaborate criticism, and a course of ingenious experiments, Mr. Davy comes to the conclusion that his former opinions concerning the metals of the fixed alkalies are accurate, and that potassium and sodium can with no more propriety be considered as compounds than any of the common metallic substances.

In the progress of chemical science surprise and pleasure often arise from extraordinary results, and the apparent elucidation of obscure and involved principles; but a more direct utility is found in the facilities it gives to arts, manufactures, and science. In a course of experiments made by Mr. Davy on nitrogen, ammonia, &c., though he was disappointed in the chief design, two practical facts turned up. It appeared from one that the passage of steam over heated manganese, may be applied to the manufacture of nitrous acid: from the other, that the ignition of charcoal and potass, and their exposure to water, may be advantageously applied to the production of volatile alkali, where fuel is cheap. These details may be deemed beneath the dignity of philosophy; but let the philosopher reflect, that from discoveries like these, the comfort, the influence, and the ease of mankind results. An elaborate analytical enquiry into the several varieties of British and foreign salt (muriate of soda,) with a view to explain their fitness for different œconomical purposes, has been instituted by Dr. Henry. In many points this is an interesting enquiry. To the medical philosopher it cannot be indifferent. The quantities of muriate of soda, daily and hourly consumed in the preparation of food, and much of which is taken into the system with that food, cannot but be believed to operate on the actions of the animal structure, and affect its stamina. To the political œconomist's it is not of less importance. An opinion has very generally prevailed both in this and other countries, that British salt is not so perfect a preserver of animal substances as that procured from France, Spain, Portugal, and other warm climates, where it is prepared by the spontaneous evaporation of sea water. The object of Dr. Henry is to ascertain, whether this preference of foreign salt be founded on actual experience, or be merely matter of prejudice. The enquiry has led to a demonstration that the British salt has a greater degree of chemical purity than the foreign salt; and the opinion of its being less applicable to œconomical purposes than that of France, Spain, &c. is a prejudice unsupported by chemical investigation or by experience.

Though it does not appear that chemistry has discovered any substance that approaches to a cure for calculus of the human bladder, yet as this science has explained the component parts of human calculi, and shewn that where certain materials predominate in the urine calculus is formed, or that the urine will, in the *diathesis calculosa*, evolve some principles that constitute calculus, it may still be hoped that a prophylactic, if not a cure, may be found. An assumption by Mr. Home, that he had discovered that liquids pass from the cardiac portion of the stomach into the circulation, led him to consider that the greater number of nephritic complaints might possibly be prevented by introducing into the stomach such substances as are capable of preventing the formation of uric acid. If the concatenation is not clearly seen between Mr. Home's opinion, that fluids pass into the circulation from the cardiac portion of the stomach, and the formation of uric acid; it must be allowed, that if by any invisible chain, this opinion has led to the discovery of a prophylactic for calculus, it has performed in 1810, what in 1808 it could not have been supposed to be capable. The substance employed to subdue the formation of this acid was Magnesia; which was found to diminish the uric acid in a much greater degree, than the alkalies when even liberally used. Taken in the quantity of a dram in the day in water, it not only diminished the quantity of uric acid, but relieved the patient of unpleasant sensations; and in one instance seems to have suspended the attacks of gout. The muriatic and nitrous acid have been likewise used with much effect as lithontriptics. These completely relieved the symptoms, and the urine deposited a sediment, in 100 grains of which was found 80 of uric acid.

The publication of the Pharmacopœia of the Royal College of Physicians in London, in 1809, had induced attention to the science of pharmacy and to the Materia Medica in an unusual manner. Numerous observations, remarks, and critiques both friendly and hostile to that production, have appeared. To examine, or even to give a descriptive catalogue of the whole of them, whether detached essays, cursory observations, laboured criticisms in Reviews, or volumes expressly published on the subject, would occupy more time and space than this Report can allow. The Essay say by Dr. Bostock, and the elaborate examination of the College publication, in the London Medical Review, are of sufficient importance, however, to be distinguished from the mere productions of the day. The nomenclature of the Pharmacopœia Londinensis has excited more observation, than its compositions, or the improvement of its Materia Medica. But with what propriety so much labour and time have been bestowed upon this part of the subject may be questioned. Precision in language, though a point of no mean importance, is secondary to the collecting and selecting substances for the cure of diseases. However energetic, terse, and perspicuous the diction of our Pharmacopœias, unless they possess the best and least exceptionable materials for the relief of the sick, they "become as sounding brass." In addition to the productions expressly professing to examine, correct and amend, numerous translations of this work have appeared under the denomination of Conspectuses, Synopses, &c. &c. &c. These have, necessarily, various degrees of merit. That published by Dr. Powell, with the concurrence and almost under the sanction and authority of the College, was not without considerable defects, which have been criticized with much severity. The conspectuses of Dr. Greaves and Mr. Todd Thompson, combining with the London, the Dublin, and Edinburgh Pharmacopœias, afford compact and useful compendia of Materia Medica and Pharmacy. Among the works which treat generally or systematically on this subject, a system of "Materia Medica and Pharmacy," by Murray, claims particular notice, both from the reputation of its author, and its own intrinsic properties. In its present enlarged and improved form, it has but little resemblance to its original state.

The introduction of new substances into the Materia Medica, or the discovery of new properties in substances heretofore employed, create an interest which must not be looked for in novel arrangements, the remodelling of language, or the melioration of technology. The period comprehended in this Report has furnished facts of this kind. A narcotic plant of the genus DATURA has been long known to possess properties of extraordinary potency. Under the popular term Thorn-apple, or the more scientific, if not classical word, *Stramonium*, it had been frequently employed both for nefarious and medical purposes. Various nervous affections had been attacked with it, and the adventurous *Storck* attempted with it to remove epilepsy and mania. The empiric found in it a ready instrument, and curious relations are given of its effects when in the hands of common thieves and and robbers.* Under suspicion or neglect, it remained, as far as regards the regular physician, an incumbrance or a disgrace to the Materia Medica. When stigmatized or forgotten as a remedy, it arose into fame suddenly, like the phœnix from its ashes. Accident, or some obscure tradition, early in the year 1810, induced some persons afflicted with disordered respiration connected, as frequently happens, with much distressing morbid sensibility, to employ this plant as tobacco is smoked. In some instances the result was sudden and almost magical. The morbid sensibility was appeased, and the nervous patient, if he was not cured, had drank oblivion of his cares, and fell into a quiet somnolency. As the charm dissolved the magic calumet was at hand to renew it. And thus from time to time the sick man smoked and was relieved: and thus from time to time his remaining energies were benumbed, until fatal coma released him radically from his sufferings; or nature, struggling with epilepsy, shook off the insidious remedy.

This history of the effects of Stramonium is here inserted, not to forbid the use of this powerful and valuable substance, but to caution the invalid against its indiscriminate and empirical employment. If instances of its injurious and even fatal effect, when smoked in some idiosyncrasies are on record; there are others where its benefit has been marked and incontestible. It becomes then the duty of the physician, from a close and attentive observation of facts as they arise, to ascertain the exact accompanying phenomena when it has either been useful or injurious, and to lay down the *ratio symptomata* that indicate or forbid its use; so that it may be known by induction when it may safely be employed, and when it should be rejected. This, as it regards the public, has become the more requisite, as empirics, availing themselves of the soothing qualities of this plant, have used many artifices to force it upon those who are suffering from difficulties of breathing, under the general term of asthma; and are

* The seeds of the *Datura metel* are still employed in India for the same licentious purposes, that they were in the time of Acosta and Rumphius. The records of the native courts of justice establish this fact. Dr. Sims has stated, that the root of the *Datura ferox*, prepared by drying and beating it into fibres resembling coarse hemp, is smoked as a specific for asthma at Madras. This account the Doctor received from General Gent, who presented to him some of the prepared root. If Dr. Sims is rightly informed as to the plant, it seems that two species of *Datura* are employed in India. General Gent is lately dead, and there are some circumstances connected with the last period of his life, that should induce caution in the employment of Stramonium.

labouring labouring to create a belief of its safety and infallibility. Though the *Datura stramonium* does not in any degree possess specific properties, never going further, as it has hitherto been used, than to relieve a symptom; yet its powers are evident enough to entitle it to an unprejudiced trial.*

It is remarkable that two diseases, which have been so difficult of cure as to be denominated *opprobia medicinæ*, should both have been conquered by specifics, say their patrons, which were discovered or brought into notice in 1810. Asthma has had its Stramonium, and gout its Eau Medicinale. Long ago, in some remote province of France, an officer whom nobody knew had the good fortune to discover properties in a plant, before unknown to all botanists and pharmacopolists; these properties manufactured with all the skill in pharmacy a military officer in the service of the King of France could possess, turned up, in the chapter of medical accidents, a specific for the gout. In the land of its nativity, this wonderful Eau Medicinale d'Husson, was discovered to be a dangerous remedy nearly related to a poison, and the sense and discretion of the government prohibited its use. Thus cut off, as was supposed, soon after its discovery, it remained long in obscurity; nor until the preux Chevalier d'Husson, in conjunction with some eleve of the charlatanic fraternity of France, had employed many expedients, was it again brought into notice. But England, not France, has been the stage of its golden triumphs. John Bull, who has much money and much gout, was the object of the benevolent d'Husson, or the equally benevolent proprietor of his secret, M. Chardron. To ease him of his pains, and—of his money, was believed by many simpletons, to be the only motives for establishing a depot of the Eau Medicinale in London. So useful, however, was it found to be to the

* In forming an estimate of the degree of hazard or safety connected with the use of this plant, it will be an essential precaution to determine the power of every individual parcel of it. It is certain the plants differ exceedingly in the degree of medicinal power they possess: perhaps ten times more can be used of one plant than of another. The preceding volume (the 25th) of this Journal contains an Essay on Stramonium, claiming particular regard both for its research and practical observation. Since the preceding part of this note was written we have seen a pamphlet on Stramonium by the editor of the Monthly Magazine, which collects into one point of view the evidence in favour of smoking that plant. We can readily admit the benevolent intention of the author, but we would moderate expectation raised too high. The history of medicine presents many instances of specifics falling into disuse, not from caprice, but from their want of efficacy.

British arthritics, that counterfeits were soon abroad; and a person, from the purest motives, advertised the public that his was the true warehouse, and he the true *Sosia*. But this is an abstruse point of speculation best settled between M. le docteur Desgenette and the Chevalier d'Husson. Notwithstanding the equivocal circumstances accompanying the first appearance of the Eau Medicinale in this land of promise, and the direct or indirect charlatanism of all its panegyrists, it well deserves the serious attention of the regular faculty. The effects produced by it on the human system are strongly characterized. In some idiosyncrasies it is inert as water, in others it proves a drastic evacuant, inducing extreme prostration of strength even to the verge of dissolution, and more than once has been destructive of life; while in another class it slowly and silently destroyed the tone and energies of the stomach and digesting organs. In the first and the last it either left the arthritic paroxysm untouched, or the diathesis remained with its train of distressing sensations, unable, under the induced debility, to appear in its accustomed active form. But there are to be added to these many cases in which it evidently relieved the disease, if it did not perform a radical cure. In these relieved, or as some have flattered themselves, cured cases, it is a desideratum to know with the most minute precision, what where the appearances consequent on the exhibition of the Eau Medicinale. Was the painful stadium of the disease removed by it without any visible operation? In the cases of its success, was there a quick and active emptying of the system by the stomach, the bowels, or the skin; or by each of these emunctories in succession? Did it act like opium and other narcotics? Or had it a peculiar, specific, and undefined operation, "miraculous," as one of its panegyrists determine it, "alleviating the torments of the gout as if by enchantment?" It is the interest of d'Husson, or the persons who have purchased the secret of the Eau Medicinale, to hide its composition in "clouds and darkness." The *auri sacra fames* is the only feeling that touches these men. But why does a member of the Royal College of Physicians of London, who has written a pamphlet, ambiguously panegyrical, on this Eau Medicinale, conceal the secret so profoundly? Because he does not himself know that secret: but his negative catalogue is very complete. It contains no metallic or mineral substance; it is not an infusion of gratiola; it is not a preparation of euphorbium, veratrum, hyoscyamus, belladonna, digitalis, elaterium, &c. &c. (p. 9.) In short, it is not made from any material hitherto known to possess properties, medicinal when employed by intelligent persons, but deleteridus rious when in the hands of quacks. More has been said of this nostrum, possibly, than it deserves. But as it is made, incontestibly, from a substance of great but uncertain activity, has relieved some cases of gout, and been fatal in others, these remarks did not seem improper, both on the principle of precaution, and with the hope of inducing varied trials with substances producing similar effects on the animal system. As far as experimental enquiry and conjecture have yet gone, the evidence seems most in favour of the Eau Medicinale being a preparation from some of the species of *Nicotiana*.

The fatal termination of Cancer, and the difficulty with which diseases of the skin are managed, especially in the form of Elephantiasis and Lepra, renders every medicine of importance which promises to relieve them. Some years ago, the public was amused with an account of a disgusting remedy employed in South America, with good effect, it was said, in these diseases. Dr. Gourlay has brought this remedy again into notice. In an account of the island of Madeira, and its diseases, written by this gentleman, it is asserted that the common Lizard (*Lacerta agilis Lin.*) is given with success, even in Cancer; and he speaks of instances of its successful employment within his own knowledge. So much has been promised, and so little has been performed upon most occasions where extraordinary and singular remedies have been proposed, that they are received, properly, with hesitation and doubt. With regard, however, to the efficacy of some individuals of the genus *Lacerta*, the testimonies of various times, collected in distant parts of the world, afford some confidence. The Greeks, the Romans, and the Arabians, had an high opinion of the medicinal properties of the Lizard, and many absurd and superstitious instances of its effects are related by the authors of those nations. Various parts of this animal were employed for different purposes, and different portions were appropriated to the cure of opposite diseases. But there seems to have been a prevailing notion with regard to the efficacy of the Lizard generally in Scrophula, and complaints of the skin; and its blood was employed as a cosmetic. Serenus Samonicus, a physician in the time of Caracalla, in a poem on medicine, observes,

"Verrucam poterit sanguis curare Lacertæ."

There is, however, nothing in the ancients on the genus Lacerta that applies to the present subject, unless their general and confused notion of some of the species being efficacious in Struma, and cutaneous defœdations, can be connected with it by a loose analogy. The moderns have examined the subject with more precision. About the year 1781 Dr. *Joseph Flores*, a Spanish physician in South America, and a member of the university of Guatimala, published a memoir at that place, and again at Madrid in 1782, in which he asserts that a specific for Cancer had been discovered in the kingdom of Guatimala. This specific was a kind of Lizard, called by the inhabitants *Lagartija*. The Indians, or aborigines of the province, were the physicians who used this remedy. Their method of cure consisted in making the patient eat, during three days, or longer if the virulence of the cancer required, the *Lagartija* prepared in the following manner. The head and tail of the reptile were cut off, the skin and entrails separated from the body, which was then eaten raw and while quivering with life. But it does not appear to be essential to give the remedy in this disgusting form*. The animal being prepared in the manner before stated, may be made into pills. Each *Lagartija* will form two pills about the size of musket balls, which are taken daily. The mode in which this remedy operates is as singular as the remedy itself. The excretions of sweat and urine are much increased, and ptyalism is excited, the saliva discharged being thick and yellow. When neither diaphoresis nor salivation occur, the defect is supplied by an ample excretion of acrid and fetid urine. When the memoir by Dr. Flores appeared in Europe it made a very general impression. The Royal Society of Medicine at Paris directed M. Carrere to examine it, and his report on the subject appeared in the 4th volume of the Histoire de la Société Royale de Medecine, with the title of Rapports sur les Virtues medicale des Lézards du Royaume de Guatimala. To this is joined another Report by Messrs. D'Aubenton and Mauduyt, on the same subject. The Spanish physicians of Europe substituted the Lizard of Spain for the *Lagartija* of Guatimala, and it was said considerable advantages had been derived from its employment. Upon this it was that the Count de Vergennes procured a

* In the St Cristopher's gazette, about 1782 or 3, it was stated that in the Leeward Islands the wood Lizard was used as a remedy for Cancer, syphilitic eruptions, &c. The Lizard is directed to be taken almost alive, the legs, tail, and head cut off, the bowels taken out, and the skin removed: or, after being thus prepared, the body being minced small, may be made into pills. These pills are given fasting, every morning about sun rise, keeping within for two or three hours after. This medicine is stated to operate by salivation, sweat, and urine, and is reported to have effected astonishing cures. The pills taken for a dose though not specified, may be conjectured to be those made from the body of one Lizard.

number number of Spanish Lizards to be brought into France, and distributed to the academicians who had furnished the preceding Reports. From the succeeding silence on the subject and the gradual subsidence of this mode of attacking Cancer, it is obvious that the remedy did not succeed with them. In Italy and Germany this new remedy did not escape the notice of medical practitioners. Dr. Flores had asserted in very positive language, that the Lizard cured cancer, syphilis, hydrophobia, and all the varieties of cutaneous disease. Fillipo Baldini, physician to the Sicilian Court, in "*Observazione sul' uso Medico de Rammari*" coincides with Dr. Flores in recommending the Lizard in all these formidable diseases. And another Italian physician Sig. Bassiani Carminate, (in *Opuscula Therapeutica)* though he does not go so far as Baldini, speaks in decided terms of its extraordinary efficacy in Scrophula. In Germany similar opinions prevailed, and Dr. Roemer in 1788 strongly recommended the use of the green Lizard: about the same time I. P. Grass published at Helmstadt a Thesis *de Lacertæ agili Linnei*. It is not a little remarkable, however, that during all this period, from 1781 to 1788, it was not distinctly known what species of the genus Lacerta it was that possessed these extraordinary medicinal properties, or what affinity the *Lagartija* of Guatimala held with the Lizard of Spain, France and Italy. But it has since been ascertained that the *Lagartija*, the Lizard gres, the Lacerta terrestris, the Langrola, and the wood Lizard, are the same with the *Lacerta agilis*.* From the period here mentioned, to the time of Dr. Gourlay's publication, the medical use of the genus Lacerta was overlooked, if not forgotten. His observations are so pointed, and his assertions so direct, that it will, probably, again be brought into notice; and the use of the Lacerta may now be either established or finally rejected.

No article of the Materia Medica has occasioned more enquiry than Mercury. In its crude state, quicksilver was once the subject of a keen controversy in this country. Its advocates pronounced it to be an universal remedy: and many

---

* The trivial name *Agilis* is happily applied to this species of Lacerta. It springs upon the minute animals on which it feeds with the quickness of thought; and on the appearance of danger, it seeks a secure retreat with equal rapidity. Far from flying from the approach of man, it seems to eye him with satisfaction, but on the smallest noise, even the falling of a single leaf, it suddenly shoots away and disappears, returns again, seems agitated, conceals itself again, returns, describes several circuitous contortions so rapidly as hardly to be followed by the clearest eye. This wonderful rapidity of motion is chiefly to be seen in warm climates, for in temperate regions its evolutions are more languid.

hundreds in London swallowed a certain portion of it daily, for a long time together. The effects produced by this practice are not detailed with distinctness, but the probability is, that it was often inert, though, in some instances, the contrary was observed; and the celebrated Barton Booth was declared to have died from its use. The exhibition of the various preparations of this excellent remedy must have produced sometimes untoward effects; but it had not been observed, until lately, that certain morbid states, distinctly characterized by specific phenomena, arise from its employment. About the year 1782, or 1783, these effects had been so far observed as to become the subject of remark, in the public lectures delivered by a very ingenious Surgeon. In 1805 and 1806 several publications appeared on the subject of an eruptive disease consequent on the use of this mineral. The disease thus produced seems to have been most frequent at Dublin, where it sometimes was fatal. Sir George Alley, a physician of Ireland, was among the first who gave his opinion on this subject to the public. The disease which Sir George Alley describes as being produced by mercury, and to which he gives the name of *hydrargyria*, is characterized by an eruption which is very variable in its appearances. In some instances there is merely a light rose-coloured efflorescence; in others, the skin presents an almost uniformly dark red tint, approaching, in a few cases, to a purple. But for the most part, the eruption appears in semi-distinct spots of a dusky red hue, which, diffusing themselves over the surface, leave but a few interstices of the natural colour. This eruption consists of a multitude of scarcely perceptible vesicles, and is accompanied with a febrile irritation, proportionate to the severity of the external appearances. The symptom which soonest produces distress, is an affection of the lungs. In some instances there is a disturbance in the respiration alone; but in others, a hard, harassing cough, and a fixed pain in the chest are superadded: and sometimes bloody expectoration. In these cases the pulse was hard as in pneumonia. Pain in the head, or violent delirium were seldom observed. Soreness of the throat and fauces was often a distressing symptom, and continued many days; hoarseness and extensive sloughing of these parts also occurred. The tongue, early in the disease, has an extraordinary degree of whiteness, but toward the close becomes parched, and black in the centre. The different degrees of *hydrargyria* are distinguished by the terms *mitis*, *simplex febrilis*, and *maligna*. Formidable this disease must have been in Dublin, for nearly one in five died. Beside this vesicular disease, under the denomination *hydrargyria*, other morbid effects have been produced produced by Mercury. These, from their resemblance to Syphilis, have been called *pseudo-syphilis*, and *cachexia syphiloidea*. These differ much from the *hydrargyria*. That is an acute febrile affection, these are chronic; and though their characterizing marks are well known, Hunter, Pearson, and Abernethy hesitate to pronounce them mercurial. Mr. Andrew Mathias, in an express treatise on the subject, has described their symptoms with more clearness and precision than had before been done; but he has not satisfactorily explained their cause, though he looks for that cause to Mercury. In the history of this mineral a fact of more importance has not occurred, than one which accidentally arose in the spring of 1810. In the month of April of that year, the Triumph man of war, took on board 30 tons of quicksilver, contained in leathern bags of 50 pounds each. These bags were picked up on the shore of Cadiz, from the wreck of two Spanish line of battle ships, lost in the storm immediately preceding the above date. The collected bags were stowed below, in the bread room, after hold, and store rooms forward; they were saturated with sea water, and in about a fortnight many of them decayed and burst, and the mercury escaped into the recesses of the ship. At this period, bilge-water had collected, the stench of which was considerable; and the carpenter's mate, in the act of sounding the well, was nearly suffocated. The effect of gas escaping from *bilge-water* is manifested, by its changing every metallic substance in the ship black. But in this instance metals of every kind were coated with quicksilver; and an affection of the mouth took place, very generally, among the men and officers to a severe degree of ptyalism, in upwards of two hundred persons. This history having been submitted to Dr. George Pearson, so well known for his chemical acquirements, he observed upon it, that " from well established principles, as well as analogies, a very reasonable explanation may be given of the effects attributed to 30 tons of quicksilver, exposed on board the Triumph in bilge-water, in a hot climate, in the beginning of Summer. The stinking gas, which was sulphuretted and perhaps phosphuretted hydrogen gas, mixed with carbonic acid, and, possibly, other gases compounded by the putrefaction of animal and vegetable matter. The deadly suffocating effects of which gases are fully ascertained; tarnishing of metals, especially silver, at a great distance, even when mixed with a large proportion of fresh air, is a well known effect of sulphuretted hydrogen. These last mentioned effects are attributable to the gases of putrefaction independently of quicksilver. But when the influence of so large a body of this metal is considered, it will be easy to account count for the whitening of metals, and the salivation of many persons in the ship. The quicksilver would rise, united or suspended by the above gases, or be even evaporated by the heat of the ship, in the common fresh air. This metal, thus suspended or dissolved, is very likely to penetrate the human body, and act upon it like the fumigation with mercury; but sulphuretted hydrogen dissolves the metal, and of course would carry it wherever the gas was transmitted."*

The Materia Medica has received important additions from a valuable catalogue of Indian medicinal plants and drugs, by Dr. Fleming. Many of these articles are yet only known in India; but the information now given will, probably, bring them into the European practice. A few only can be here noticed. In running over the alphabetic arrangement, the *Asclepias asthmatica*, a plant growing in the northern Circars, but not in Bengal, first deserves regard, as affording, possibly, a substitute for ipecacuanha; and possessing, perhaps, some properties different from that plant. It is employed in dysentery and asthma with great success, by the Hindu physicians. This species, of a very numerous genus, was discovered in the woods of Ceylon, by the late Dr Kœnig (Linn. suppl.), and to him is the public indebted for all that is at present known concerning it. In the examination of the properties of the asthmatic swallow-wort, it would be unwise to overlook its thirty-three known brethren, among whom may perhaps be found individuals possessed of similar virtues. The *Boswellia thurifera* furnishes the gum-resin olibanum, which is yet given (Phar. Lond. Edit. 1809) to the *Juniperus lycia*. *Cœsalpina bonducella*, a native shrub of both Indias, but known as a medicine only in the East, produces seeds intensely bitter, and possessing tonic powers in a very high degree. The Hindu physicians employ these seeds as a general tonic; and particularly in intermittent fevers, for which they are considered as an almost infallible remedy. The *Calcaranja*, the Hindoo name of this plant, is most efficacious when used with a decoction of the *Gentiana chirayita*, another valuable plant, indigenous in the mountainous countries north of the Ganges. *Carica papaya*, (a native of South America, but

* As opinions are much at variance on the rationale of this occurrence on board the Triumph, it is hoped that a further investigation will take place. We know that Dr. Baird, to whom the public is indebted for the above statement, and who pursues, with an indefatigable spirit, every object calculated to benefit physical science and improve our medical naval œconomy, is endeavouring to effect a full and satisfactory explanation of a phenomenon so interesting to philosophy as well as to medical science.

which has rapidly spread, from the Phillippines and Moluccas to which it was brought by the Spaniards, over all the countries of India,) gives a milky juice from its fruit on incision, which the inhabitants of Bourbon and the Isle of France consider as the most powerful vermifuge yet known. The seed of the *Osimum pilosum*, the *Rihan* of the Hindoos, has the property of furnishing a mucilage, when infused in cold water, which is used by the natives as a demulcent in catarrh; and is also a favorite medicine with the Hindoo women, for relieving the after pains of parturition. The seed of the *Beloneia officinalis* of our woods has the same quality. When a short time infused in cold water, every individual seed of the *Betony* is surrounded with a thick coating of mucilage. The bark of the *Punica granatum*, was found effectual in dislodging the tænia. The extraordinary property which the seed of the *Strychnos potatorum* possesses of clearing muddy water, and rendering it palatable, has claimed a place for it in this catalogue. This property is so well known to belong to this seed, that it is called by the English in India, clearing-nut, and is sold in every market for this purpose. The clearing-nut is constantly carried about by our more provident soldiers in time of war, to enable them to purify their water. The leaves of the *Vitex trifolia* are employed by the native Hindoos as a discutient. Their efficacy in dispelling inflammatory swellings of the joints from acute rheumatism, and of the testicle from supressed gonorrhœa, has often, says Dr. Fleming, excited my surprise. In the *Meloe cichorei*, which abounds in every part of Bengal, Bahar, and Oude, has been discovered a substitute for the Cantharis. It has been found to produce effects precisely similar to the officinal blistering fly: Lytta vesicatoria, P. L. 1809.

Among the newly discovered properties in medicinal substances, the almost specific one in the *Oleum Terebinthinæ*, against the Tape-worm, deserves particular regard. It has now been given in a great number of cases with much success, and is apparently so free from all hazard, that its character in this complaint may be considered as established.

The white oxyde of Arsenic, which is now admitted into the London College Pharmacopœia, has had its operation on organized bodies critically examined in an inaugural dissertation, *de Effectibus Asenici*, &c. by Dr. Jaeger. This potent mineral appears to be a certain and quick poison to plants at every period of their growth, and that it destroys them by a chemical action which exhausts the irritability of vegetable life. The irritability of the *Mimosa sensativa* was exhausted before the plant itself was destroyed. When applied plied in a destructive quantity to animals, their death was preceded, in every instance, from the infusory animalculæ up to the man, by inordinate motions, and a most remarkably increased secretion of lymph from the mucous membranes. Frequent fluid stools took place in all classes of animals ; the respiration of those animals which breathe by lungs, became difficult and laborious, and warm blooded animals experienced extraordinary thirst. In birds and mamalia, frequent violent vomiting took place, and commonly was the commencement of the scene to which convulsions put an end. Rabbits, which ruminate, did not vomit; dogs and cats gave the first indication of their uneasiness by a change in their voice. The appearances that arise from a poisonous dose of this mineral, both during life and after death, are minutely examined and detailed. And from a very full view of the subject it is concluded that Arsenic does not act mechanically by the sharpness of its particles, nor by its local action on the stomach, nor in the manner of acrid poisons or dephlogisticating substances, nor upon the nervous system ; but is analogous to the poison of the viper and ticunas, which act primarily upon the blood. In these very minute inquiries into the action and operation of Arsenic as a poison, it does not seem that Dr. Jaeger has made the report of this mineral being the efficient material in the *Acqua tofana* at all the subject of his investigation. The proofs of poisoning by Arsenic are of two kinds: pathological, derived from the symptoms which precede death, and the appearances of the body after death: chemical, derived from the properties of the mineral. While this dissertation examines, arranges, and analyses the phenomena that accompany the operation of this virulent substance, a French physician, M. Casimir Renault, has presented the faculty with *Nouvelles Expériences sur les Contre-Poisons de l'Arsenic.* This writer considers vomiting the surest remedy for poisons taken into the stomach, and which can act only by bringing away the deleterious material. He has, however, an ingenious substitute. He proposes, with a syringe, to which is affixed a pipe long enough to pass into the stomach, to wash that organ completely. A quantity of any fluid may be thrown into the stomach with this syringe, and again drawn out with the same instrument. The repetition of this as frequently as may be required, will dilute, dissolve, and extract the poison. How far this *Lotura ventriculi* will answer in practice remains to be determined.

Of the three branches into which the profession of medicine is practically divided, after the ground gone over, not much remains to be said. Neither Midwifery, Surgery, nor the the practice of Physic, have, however, been without improvement or innovation, during the year 1810. The pages of this Journal, in that period, have given some discussions on the practical parts of Midwifery, particularly respecting the management of twin cases. But Dr. Merriman's Dissertation on Retroverted Uterus and extra Uterine Fœtation, affords the principal novelty in this department of the healing art. The ingenuity of that Dissertation has been fully admitted, notwithstanding the impossibility of some of the writer's conclusions being true.

The rapid progress which the operative part of Surgery is making toward perfection, excites a double sentiment in the reflecting mind: admiration at the excellence to which the operative part is reaching, and apprehension lest this excellence should, at times, lead to its employment when milder methods might succeed. It is not to be disbelieved that as Surgeons become assured of the facility and certainty of their operations, these operations have sometimes been needlessly resorted to; and that mutilations have occasionally taken place as much for the honour of the Surgeon as for the benefit of the patient. While operations were more difficult, and their results more precarious, the Surgeon hesitated and the patient recovered. The per-contra of this, however, preponderates in the scale of good. Diseases which once were fatal, whether left to their natural course or operated for, are now cured, with great certainty, by new operations, or by those previously known. Aneurism presents an instance. For some forms or localities of this disease, Surgery, a few years since, had no remedy. Aneurism of the femoral artery, when seated high up on the vessel, was irremediable. Aneurism of the subclavian or carotid arteries, was considered as beyond the reach of art. But these are now operated for with success.

If mechanical surgery is thus highly improved, can it be allowed that the reasoning part has been equally progressive? In this age of philosophic deduction, strange would it have been if this spirit had in no degree reached the Surgeon. Satisfied, however, with the precision and dexterity of his mechanical acquirements, the Surgeon has fallen, less frequently, into the *deliramenta doctrinæ* than the other branches of the medical profession. But it must not be forgotten that Mr. Abernethy, learnedly expert in the mechanical part, has gone deeply into the rationale of the science; and, though influenced by a shade of hypothesis, has preserved to modern surgery that decided character for investigation, which was imparted to it by John Hunter. A republication of Mr. Abernethy's works, treatises by Messrs. Whately, Copland,

 Ramsden,

Ramsden, Geohagan, Wadd, Bell, &c. &c. have improved the practice in some instances; in others have either described new diseases, or placed in a new point what has before been investigated. Mr. Whately's disease in the Tibia, as the sequela of Fever, must be believed, until this ingenious Surgeon farther elucidates the subject, to be a form or variety of *Necrosis*. The history and treatment of diseases of the Rectum and Anus, have been explained with precision by Mr. Copland; who has combined erudite illustration, and practical facts, with a close observance of nature. The distress and irritation which hæmorrhoidal excrescences communicate to the whole frame, has induced surgeons to extirpate them, either by incision or ligature. Both these methods have been accompanied or followed by untoward terminations. Dangerous hæmorrhage, or fatal inflammation resembling that which occurs in incarcerated hernia, are always possible events, and have been the consequence of those remedial attempts. To avoid the hazards of either of these, it is proposed to bruise the stem of the hæmorrhoid, in a manner similar to that which animals employ on the funis umbilicalis; and thus prevent hæmorrhage, or that inflammation which has extended to the intestinal canal. To effect this with certainty a correspondent advises the use of a forceps, sharply (perhaps prominently) dentated. Mr. Ramsden has referred many of the diseases of the Testicle and its tunics, to a peculiar state of the Urethra, as their cause; and has described, as he conceives for the first time, some circumstances in that canal, operating very powerfully, to excite or to exasperate almost the whole of the morbid states of that gland. By a proper application of the bougie to the urethra, he states, from actual observation, that many disorders of the Testicle may be speedily removed, which under every other kind of management are either tedious or dangerous. A branch of Surgery, almost new to this country, and which but a few years since was wholly in the hands of foreigners, is now applied to with a degree of attention which assures to the English Surgeon an equality, at least, with those of the continent. The delicate structure of the organ of vision, the numerous diseases to which it is liable, resulting, perhaps, from this delicacy of structure, and the increasing catalogue of these diseases, combine to make this branch of Surgery of great importance. The establishment of institutions for treating the diseases of this organ, the numerous publications, and the success with which operations, once unknown or thought impracticable, are performed, shew distinctly the progress of knowledge on this interesting subject.

There is nothing connected with medical science that involves, volves, more seriously, the interest of society and the credit of the faculty, than Vaccination. It has had to encounter, very fully, the difficulties that all new modes of treating diseases, and all projects for securing the health of mankind, undergo. From a variety of motives and feelings its progress has been obstructed. A few persons have had a conviction of its insecurity as a preservative from small-pox: some have been prejudiced against the discovery itself, or against the promoters of the practice: but more have resisted with the view of raising themselves from obscurity, or to insure a lucrative employment by spreading variola. Under all these disadvantages Vaccination is establishing on the firm basis of experience. Its clamorous and injudicious friends are tired into silence, and leave the defence of the practice to its own merit, as displayed in a numerical logic, of all eloquence the most persuasive. It has been seen, in authentic documents, that every method of encouragement has been taken by the government of France to promulgate this discovery, and the infant heir to the monarchy has undergone Vaccination. In the island of Ceylon 25,697 were vaccinated in 1809, making a total of 128,732 persons vaccinated there from the period of its introduction; and the small-pox is said to be exterminated. The Report of the National Vaccine Establishment in London, made to the House of Commons in last March, states, that no failure in any of its stations has occurred in any case of Vaccination. In the Royal Military Asylum, containing 1,100 children, where Vaccination has been employed since 1803, but one child has been lost by small-pox, and that was not vaccinated, on the presumption that it had previously passed through *variola.* In the Foundling Hospital no death by small-pox has occurred since 1801, when Vaccination was introduced. From Manchester and Glasgow similar successful returns are made. In Dublin and Edinburgh the Report states that Vaccination is becoming general, and with a success equal to its extension. It is the duty of the Historian to record the strong prejudices still existing against Vaccination; and that a few unfortunate cases keep alive and increase these prejudices. But though it would be unfair to deny that cases of small-pox after Vaccination have sometimes happened; yet the proportion that these unfortunate cases hold with the secure ones is so small, as hardly to furnish an objection to the practice.

In the theory or institutes of Medicine, the character of the favourite hypothesis has been so frequently drawn from the peculiar habits or pursuits of its projector, as completely to justify the observation of M. d'Alembert. "La philosophie prend la teinture des esprits ou elle se trouve. Chez un mé-

 taphysicien, taphysicien, elle est ordinairement toute systematique ; chez un géomètre, elle est souvent toute de calcul." It is not, abstractedly, of much importance that the medical theory of the chemist be chemical, or of the mathematician mathematical. But the general principle so frequently and so far influences practice as to lead to the most dangerous errors. At one period stimulants and tonics were to be employed in all cases, at another evacuants ; now an acid was to be encountered, now an alkali. All these hypothetical opinions have been acted upon as earnestly as if they had been erected on the foundation of immutable truth. When it was observed in the view of the progress of medical science in 1808, that the tide of opinion was strongly setting against the stimulating therapeutica of the Brunonian School, it was not expected that occasion would soon be given for lamenting the opposite and more dangerous extreme. John Brown reckoned the pure sthenic diseases to be few in number; and even these were pronounced prone to run into indirect debility, and then to fall into his great class, asthenia. Thus were his scholars taught to fear the sud denfailure of the *vis vitæ*, and to guard, even whilst the inordinate action continued, against the impending debility. It is, indeed, surprizing that this hypothesis, especially as indirect debility was a term in constant use and defined with accuracy, did not lead to different practice. It might have been presumed, as the pupils of this school were taught that the subsequent debility was proportioned to the previous increase of action, some means would always be taken to moderate that increased action : or, at least, that stimulants would seldom be employed, while it continued, to obviate the dreaded debility. But it was left to the practitioners of the present time to see the force of this inference and its application to practice. If any individual were to be fixed on as having been most instrumental in effecting this change, Dr. Hamilton, the author of the treatise on the use of purgatives, would be the man. That treatise, full of practical knowledge, and of clear deductions, spoke conviction. Many other practitioners may, however, claim a share in this important change. *Scarlatina*, long thought to have a close affinity with the asthenia, had, for many years, when it assumed what has been called a malignant aspect, been treated with cinchona, wine, opium, alcohol, æther, &c. &c. Under this method its fatality was most serious. Families of children were swept away. In these instances the practice correctly quadrated with the principle : but the principle was, probably, erroneous. In the 25th volume of this Journal a history of the progress of *Angina maligna* in an eastern district of the kingdom has been recorded.

There

There it is shewn, on respectable authority, that nearly the whole of those who were treated with tonics and stimulants died; while those who were blooded, purged, and had other means employed to restrain excessive action, with few exceptions, recovered. If the facts stated are not sufficiently distinct, as to the phenomena and characteristic marks of the disease, or are not enough explicit as to the successful method of treatment, the practitioners of Ipswich, and other parts of the county of Suffolk, who have given the detail, are imperiously called upon to shew, beyond equivocation, what the disease was in which the stimulant treatment was so generally fatal, and where the contrary was so successful.

While a cautious and rational deviation from the stimulating system of Brown claims the fullest approbation, it cannot pass without remark, that many have employed the opposite method to an enormous extreme. Dr. Robert Jackson, an army physician of great experience and observation, but influenced, possibly, by some hypothesis, has, in the fevers of camps and military hospitals, employed the depleting fashion to a most extraordinary, if not extravagant extent. Under his immediate direction the bold abstraction of blood may have sometimes preserved life; but the employment of this remedy by a herd of imitators, who, without selection, subject their patients, *en masse*, to the lancet, must create alarm. It is against all reason and observation that every soldier, suffering by fever, whatever his age or temperament, whatever the period of his disease, or whatever peculiarity there may be in the symptoms, either as to degree or quality, should equally bear the loss of 20 or 40 ounces of blood at a time, and often with quick repetition. In cases of topical inflammation this may be right; but even then it will require a nice estimation of the forces of the system, to ascertain who can bear the loss of 200 or 300 ounces of blood in four days, or who would be destroyed by such an evacuation. But in cases of fever, without local inflammation, generally either typhus, or approaching to that type, such practice must be considered as hazardous, and, possibly, will often be fatal. If this practice should ever have been resorted to in the last stadium of these fevers, or should have gone into the period of convalescence, it will appear more rash, wild, and fatally extravagant.

Professing to give an "abstract and brief chronicle" of the progress of the healing art, to have passed over unnoticed, even a report of a practice which reduces science to the rude efforts of uninstructed barbarians, would have been a dereliction of principle. If, in our naval and military hospitals, this extraordinary practice has prevailed, it cannot long continue.

tinue. The majority of army and navy surgeons are known to be humane and scientific; to add a perfect knowledge of the structure, and a profound acquaintance with the functions of the frame, to cautious practice. If, for a period, many of these should have fallen into this irrational proceding from the influence of superiors, or from the imperfection of human nature, they will soon return to methods more consonant to common sense, and the true principles of medical science.

In the walks of private life the most absurd hypothesis has its poison confined within narrow bounds; its objects are, comparatively, few, and it is restrained by public opinion: but in fleets and armies, and in military hospitals, uncontrouled by fear of the world's censure, and even concealing, very generally, the rotine of practice, theory may be carried to the most destructive length. Then will human life be sacrificed in a ratio agreeing with the error of the hypothesis, and the extent of its application; and the physician must forego his claim to the sentiment of *Cicero*. HOMINES AD DEOS NULLA RES PROPIUS ACCEDUNT, QUAM SALUTEM HOMINIBUS DANDO.

*Princes Street, Cavendish Square, June 30th,* 1811.

---

*To the Editors of the Medical and Physical Journal.*

