# Corrigenda

**Published:** 1811-07

**Authors:** 


### *CORRIGENDA.*

P. 31. For *Betoneia*, read *Betonica*.

