# Corrigenda

**Published:** 1811-08

**Authors:** 


### *CORRIGENDA.*

Page 16, line 19, for *thinner* read *thicker.*

*Printed by E. Hemsted, Great New Street, Fetter Lane.*

